# Home Industry and the Sweating System

**Published:** 1896-06-27

**Authors:** 


### MODERN SOCIOLOGY.

### HOME INDUSTRY AND THE SWEATING SYSTEM.

WE have before us two reports dealing with the same question, but hailing from the two sides of the Atlantic, which well deserve comparison. The first is an article on the Sweating System, which appears in the "Bulletin of the Department of Labour" for May, 1896, issued from the Government Press at Washington; the other is a report on the Swiss House Industries, which appears in the June number of the *Economic Journal.* As some sort of definition, it may be said that by the "sweating system" is meant that condition of labour in which a maximum of work in a given time is performed for a minimum of wage, and in which the ordinary rules of health and comfort are disregarded. It is inseparably associated with contract work, is aggravated by sub-contracting, and shows itself in its greatest intensity where work is taken home and dwelling-houses become workshops and children operatives.

Wherever there is a large foreign population, people who from their being strangers and of a strange tongue are more or less helpless and at the mercy of others, where these foreigners have been brought up in habits of exceeding industry and careful thrift, and accustomed to a low standard of living, and especially where the industry in question is one which is comparatively easy to learn, so that its ranks are recruited from large numbers of people, there the sweating system thrives. One poor starving creature competes with another for work; what one refuses another immediately jumps at; and thus the remuneration for labour is reduced to the lowest point compatible with the maintenance of life on the lowest level acceptable even to the least exacting.

At this remuneration the strongest, by working hard and submitting to privations, can just live, while the weaker members are forced by competition to take what they can get or go utterly to the wall. Needless to say, this is individualism as it shows itself in its most repulsive form, and also that this form of individualism, while held up by those who advocate the organisation of labour as an example of the miserable end to which all will come who refuse to accept their tenets, is also the thing they mostly dread as interfering with their projects.

The conditions which make the sweating system possible are: first, crowded populations in large cities; second, high rent; and third, contract work. Probably high rent is the cause of its greatest evils in large cities. Where rents are lower the shops are larger, and the evils are not so acute; but where rents are high the tendency is to use the same room as kitchen, bedroom, and workshop, and thus, while saving in rent, to sacrifice the health of the workers and to spread disease among those who use the articles they make.

The bottom of the evil is, on the one hand, the petty contractor, on the other the absence of any other means of livelihood. The large employer of labour, who has built great factories, has at least given hostages to his workpeople that he will deal decently by them. He has sunk his money, and can only get interest upon it by the help of his workpeople, who thus become essential to him to such degree that they may be almost said to become partners in the concern.

The petty contractor, however, makes a great difference. When he arrives on the scene the manufacturer no longer comes in contact with the actual workers; they only know the sub-contractor. To strikes he is indifferent. He has not sunk money in land, or mill, or machinery, and if he choose to cease giving out work his expenses stop. He has no rent, no rates, no wages, no interest to pay; he has but to wait for business to revive; and meanwhile the workers starve. They are wholly dependent on the masters for their supply of work, and thus they become their veritable slaves.

Let us now turn to Switzerland, where, throughout the land, there is to be found a remarkable system of house industry, which seems to a large extent free from these evils, an industry so remarkable that in some places machinery has had to give way to the exceeding energy of the home workers.

In wicker work, wood carving, hat making, in the manufacture of knitted goods, straw goods, embroidery, and in the watch and clock industry a very large percentage (ranging from 60 to 100 per cent.) of the workers do their work at home.

Opinions as to the ultimate effect of these home or house industries may vary, and no doubt some are ready to say that their tendency is to keep down wages, *i.e.*, as calculated in money. But there seems to be a large amount of evidence to show that the effect of the Swiss system of house industry is to bring employment and comfort to a large number of people who without it would be on the brink of destitution, and thus in some districts at least to check the growing tendency to emigration by providing work at home.

What, then, is the difference between the home industries of Switzerland and those home industries which, like the manufacture of clothing and the making of matchboxes, in large cities, generally spell "sweating." The difference would seem to arise from this, that in Switzerland a large proportion of the people are engaged in agricultural pursuits, and that home industries become sometimes a means of filling up spare time during winter months, and sometimes a means of providing an occupation for those who, although living in the homestead, cannot find on the farm any outlet for their energies. It is to be feared that even in Switzerland, amid the beautiful scenery for which the country is so celebrated, there are large numbers of people who work for inordinately long hours; still, taken as a whole, the home industry is a supplement to other forms of work. The ideal would be that every man should have variety; that the factory worker should spend his evening on his allotment, and that the farm worker should spend his long winter at such house industries as we find so widely spread in Switzerland. It is to be feared that we are all far from the ideal. Division of labour, tying each man down to one small job, is the curse which civilisation imposes on us all. Yet, although hard work is the lot both of the sweated Jew tailor in the slums and of the laborious Switzer in the mountains, we cannot but think that, in so much as the Swiss home industries offer the more variety, the Switzer's lot is best.

